# Supplementary material for: Incidence, mortality, and predictive factors associated with acute respiratory distress syndrome in multiple trauma patients living in high-altitude areas: a retrospective study in Shigatse
Source: PeerJ. 2024 Jun 17;12:e17521. doi: 10.7717/peerj.17521 (PMC11188934; doi:10.7717/peerj.17521)
Supplement: Supplemental Information 2 [file peerj-12-17521-s002.docx]

Codebook

1. ASA：I toⅢ，1 to 3
2. Gender: male 1, female, 2
3. Smoking: yes, 1; no, 2.
4. Chronic disease: no, 0; yes, 1.
5. Pulmonary complications before surgery: no, 0; yes, 1.
6. Craniocerebral trauma Limb trauma Thoracic injuries Abdominal trauma: no, 0; yes, 1.
7. In-hospital death: no, 0; yes, 1.
